# Long‐term dosimetric stability of multiple TomoTherapy delivery systems

**DOI:** 10.1002/acm2.12085

**Published:** 2017-05-02

**Authors:** Jennifer B. Smilowitz, David Dunkerley, Patrick M. Hill, Poonam Yadav, Mark W. Geurts

**Affiliations:** ^1^ Department of Human Oncology University of Wisconsin ‐ Madison Madison WI USA; ^2^ Department of Medical Physics University of Wisconsin ‐ Madison Madison WI USA

**Keywords:** dosimetric stability, tomotherapy, Radixact

## Abstract

The dosimetric stability of six TomoTherapy units was analyzed to investigate changes in performance over time and with system upgrades. Energy and output were tracked using monitor chamber signal, onboard megavoltage computed tomography (MVCT) detector profile, and external ion chamber measurements. The systems (and monitoring periods) include three Hi‐Art (67, 61, and 65 mos.), two TomoHDA (31 and 26 mos.), and one Radixact unit (11 mos.), representing approximately 10 years of clinical use. The four newest systems use the Dose Control Stability (DCS) system and Fixed Target Linear Accelerator (linac) (FTL). The output stability is reported as deviation from reference monitor chamber signal for all systems and/or from an external chamber signal. The energy stability was monitored using relative (center versus off‐axis) MVCT detector signal (beam profile) and/or the ratio of chamber measurements at 2 depths. The clinical TomoHDA data were used to benchmark the Radixact stability, which has the same FTL but runs at a higher dose rate. The output based on monitor chamber data of all systems is very stable. The standard deviation of daily output on the non‐DCS systems was 0.94–1.52%. As expected, the DCS systems had improved standard deviation: 0.004–0.06%. The beam energy was also very stable for all units. The standard deviation in profile flatness was 0.23–0.62% for rotating target systems and 0.04–0.09% for FTL. Ion chamber output and PDD ratios supported these results. The output stability on the Radixact system during extended treatment delivery (20, 30, and 40 min) was comparable to a clinical TomoHDA system. For each system, results are consistent between different measurement tools and techniques, proving not only the dosimetric stability, but also these quality parameters can be confirmed with various metrics. The replacement history over extended time periods of the major dosimetric components of the different delivery systems (target, linac, and magnetron) is also reported.

## 1 Introduction

TomoTherapy is a helical radiation therapy delivery system developed at the University of Wisconsin^1^. There have been three major system iterations incorporating several hardware and software changes since its clinical inception in 2002. The initial delivery system was called Hi‐Art. The second generation system, referred to as TomoHDA, was capable of delivering both helical (H) and static gantry “direct” (D) treatments, as well as advanced (A) dynamic jaw planning capabilities. Radixact is the newest generation of TomoTherapy delivery systems and represents a redesign of both the gantry and the treatment planning system. The principle dosimetric change on the Radixact is its capability to treat at higher dose rate of 1000 cGy/min, as opposed to the 850 cGy/min nominal value on the TomoHDA units. The linear accelerator and the MVCT detector are the same on the Radixact as the TomoHDA.

The most significant change for the dose output stability on a TomoTherapy unit was achieved with the addition of a dose servo‐controlled system, introduced in 2011^2^. The Dose Control System (DCS) is standard on the TomoHDA and Radixact systems and can be installed as an upgrade on the Hi‐Art systems. DCS serves to improve rotational and temporal dose output stability by actively monitoring and adjusting monitor chamber and gun current to maintain set points. Additionally, the TomoHDA and Radixact systems feature a Fixed Target Linear Accelerator (linac) (FTL), whereas the older Hi‐Art systems used a rotating target. The FTL improves target life by removing the tungsten target from direct contact with the water cooling system, which is known to cause target wear and dosimetric instability^3^.

Like all clinical linear accelerators, periodic quality assurance (QA) of the TomoTherapy delivery systems is recommended as part of a quality control (QC) program. The AAPM Task Group 148 provides guidance on TomoTherapy specific procedures and tolerances^4^. Broggi et al. presented the results of a 2‐year QC program for a Hi‐Art helical TomoTherapy system without DCS^5^. They reported good reproducibility of mechanical and dosimetric performance. Other groups have investigated the MVCT detector stability^6^. Similar longitudinal studies have been performed for traditional C‐arm linear accelerator systems to monitor any changes or drift in machine output over the lifetime of the system^7^. To add to this body of work, we report on the energy and output stability of multiple TomoTherapy systems of different generations including a DCS/FTL system operating at a higher dose rate. The objective of this paper is to examine the dosimetric stability of six different TomoTherapy units over the lifetime of this technology. Changes in performance over time, and with system upgrades, are presented.

## 2 Methods

Energy and output were tracked using monitor chamber signal, onboard MVCT detector signal, and external ion chamber measurements. The systems and monitoring periods include three Hi‐Art (67, 61, and 65 months), two TomoHDA (31 and 26 months), and one Radixact unit (11 months.). Four of the systems use the DCS with a FTL: the newest Hi‐Art, two TomoHDAs, and Radixact. These systems are summarized in Table 1. Because the newest Hi‐Art system (SN 103) was upgraded with DCS and a FTL, QA results are separated into pre‐ and post‐DCS/FTL upgrades. All but the Radixact system are clinical treatment delivery systems and operate at a nominal dose rate of 850 cGy/min at isocenter. The Radixact used in this study is a nonclinical unit operating at a nominal 1000 cGy/min at isocenter.

**Table 1:** Systems studied and monitoring periods. All systems, except the Radixact research unit are clinical treatment units. The Hi‐Art SN 103 is listed twice because DCS was added and the data for this machine is considered with and without DCS

TomoTherapy unit	Monitor chamber & exit detector (mos.)	Ion chamber (mos.)	Service records (mos.)
Non‐DCS 04, Hi‐Art	67	NA	42
Non‐DCS 96, Hi‐Art	61	NA	34
Non‐DCS 103, Hi‐Art	65	NA	21
DCS 103, Hi‐Art	37	32	33
DCS 477, TomoHDA	31	27	31
DCS 488, TomoHDA	26	26	26
DCS RS, Research	3	8	11

The output stability is reported as deviation from reference monitor chamber signal for all systems, and from external ion chamber measurements for four systems. The energy stability was monitored using the relative (center versus off‐axis) MVCT detector profiles and/or the ratio of chamber measurements at two depths (PDD ratio). Re‐baselining of expected values following machine service was only performed after independent validation of a given metric (output or energy) with an ion chamber measurement. Additional ion chamber measurements were used to investigate the output stability of the RF chain over extended treatment deliveries on an TomoHDA and Radixact system.

### 2.A Internal monitor chamber and exit detector data

The internal monitor chamber and MVCT detector signals were used to assess output and energy stability, respectively. Of the two monitor chambers, only the primary monitor chamber signal (dose1) was used for this study. For the Hi‐Art systems (SN 04, 96, and 103 non‐DCS), the average dose1 signals were obtained from field service records of a weekly “J48 RotVar” QA procedure. The J48 RotVar procedure is a 200 s nonmodulated rotating delivery with the largest jaw setting (nonclinical, 48 mm nominal jaw width). After the DCS installation on Hi‐Art SN 103, the average dose1 signal during a J7 RotVar (clinical beam, 10 mm field width, rotation variation) procedure was tracked because that was the field size used for the weekly QA for this unit. The stability of the output is independent of the jaw size and only affects the relative signal magnitude for a given machine.

**Figure 1:**
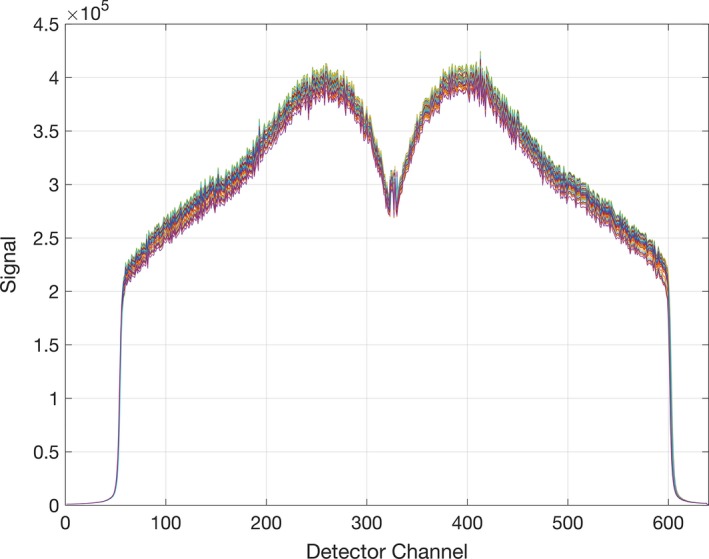
Exit Detector signals from 175 J48 RotVar procedures on SN 04. The RMS difference in the profile with respect to baseline is used a surrogate of beam energy. The characteristic shape of the flattening filter‐free beam is obscured in the center detector data due to the deliberate offset of the CT detector focal point from the beam source point leading to a lack of buildup in the center channels.

During these 200 RotVar procedures, MVCT detector data are recorded on all channels. The stability of the shape of this flattening filter‐free lateral profile is a good surrogate for beam energy stability, and has been used by other investigators for this purpose^8^. A sample of this profile data for SN 04 is shown in Fig. 1. The characteristic triangular shape of the flattening filter‐free beam is obscured in the center detector data due to the deliberate offset of the CT detector focal point from the beam source point leading to a lack of buildup in the center channels. The detector array was designed for a kilovoltage beam with a closer source. Only the central detectors are focused on the source. The result is that off‐axis detectors have more of the beam passing through the septa, which adds build‐up to those outer detectors for the megavoltage tomotherapy imaging beam. Less build‐up is provided for the central detectors, and therefore less signal.

The monitor chamber data and exit detector data were manually processed using MATLAB™. The monitor chamber output signal is reported as a difference from the average signal over the monitoring period. The extensive monitor chamber data archives included data generated prior to, and during, field service measurements. Therefore, monitor chamber signal analysis was limited to the data that were within ±3% of the average, since larger variations were from the machine being run under nonclinical delivery conditions. In addition to the monitor chamber analysis, the daily output on all machines was verified on all clinical days to be within ±3% of the baseline in accordance with AAPM guidelines^9^.

To evaluate beam energy, the exit detector central channels (80–570) were extracted from each 200 s RotVar. The normalized ratio was then computed using the first dataset as a reference. The average Root Mean Square (RMS) across each channel of the normalized ratio was then computed and reported for each run.

For the TomoHDA and Radixact, the same monitor chamber and lateral profile at the exit detector was acquired using vendor‐supplied diagnostics and analysis software, TomoTherapy Quality Assurance (TQA)^10^. The TQA software offers various modules to capture and analyze machine data. The “Daily QA” module is a 5‐min rotational procedure consisting of several tests, one of which approximates the J48 RotVar. The monitor chamber data and output ratio (relative to the calibrated baseline value) and RMS of the exit detector channels relative to baseline values are acquired and computed by the TQA software. These data were captured on each clinical day as part of morning QA.

### 2.B Ion chamber measurements

Ion chamber measurements were taken in Solid Water™ slabs as part of routine QA measurements. Exradin™ model A1SL ion chambers were positioned at various depths (1.5, 10, and 20 cm) in 15 cm × 40 cm slabs of Solid Water™. The Solid Water™ was placed with the longer dimension perpendicular to the long axis of the couch. A nonmodulated, J48 jaw width, static gantry and couch, 60 s procedure was delivered. The readings at 1.5 cm are used to track the output, and the ratio of the 10 cm and 20 cm depth readings tracked the beam energy. Ion chamber data were not available for the earlier systems (SN 04, 96, and 103 non‐DCS). The ion chamber data were acquired as part of the daily QA for SN 103 (with DCS) and monthly for the TomoHDA and Radixact systems.

In addition to routine QA, intrafraction stability of the RF chain was evaluated by delivering extended delivery time, nonmodulated plans to the Solid Water™ slab and ion chamber. This test was performed with the same field as the routine QA static check. Three different plan durations (20, 30, and 40 min) were delivered multiple times on different days on TomoHDA clinical systems and the Radixact unit. The signal was collected at 10 and 20 cm depths in solid water with the TomoTherapy electrometer and analyzed for output and energy stability. The coefficient of variation (CoV) of the output was reported for each depth to provide a measure of output stability. To quantify energy stability, the output was averaged for each 60 s of data and the ratio at each depth was calculated. The CoV of these ratios for each plan was reported as the measure of energy stability.

### 2.C Service records

Major service upgrades relating to dosimetric stability were tallied for the delivery systems in this study. Magnetron, linac, and target replacements were considered dosimetrically significant. Routine work (e.g., addition of dielectric gas), MLC system work, and mechanical repairs were not recorded. Complete service records were available for the TomoHDA and Radixact systems. The service history for the Hi‐Art systems was assembled from paper records in the clinics and service records provided by Accuray™, but unfortunately did not include the complete duration of this output and energy study. The study periods are summarized with the dosimetric study periods in Table 1 and with the service data in Table 5.

## 3 Results

**Figure 2:**
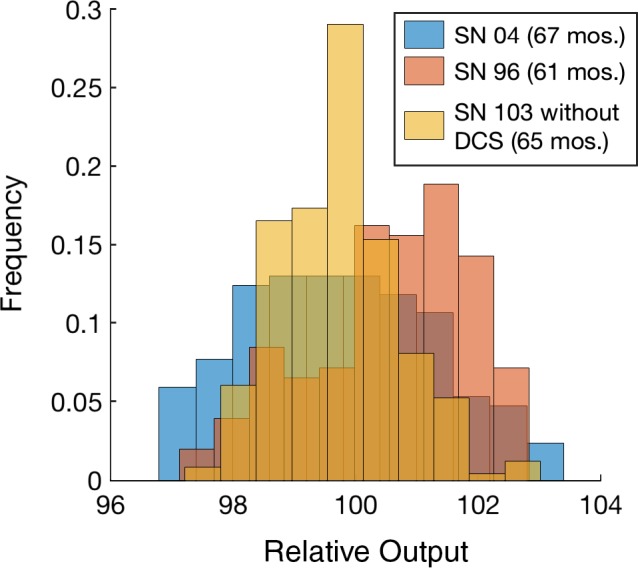
Relative output for non‐DCS systems measured by the monitor chamber**.** Data for days on which the machine was being serviced or otherwise out of the daily ±3% tolerance (and therefore not acceptable for treatment) are not included.

**Figure 3:**
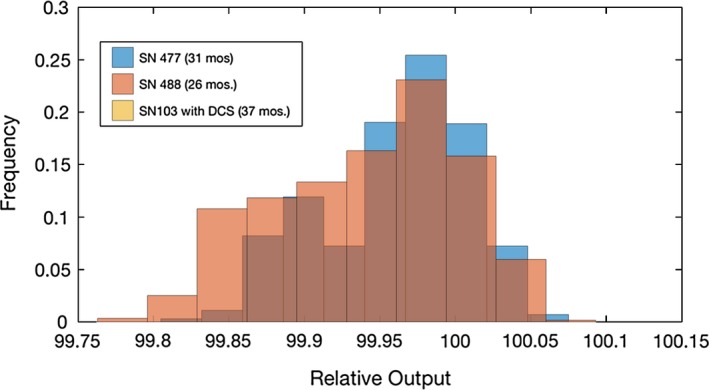
Relative output for DCS systems measured by the monitor chamber**.** Data for days on which the machine was being serviced or otherwise out of the daily ±3% tolerance (and therefore not acceptable for treatment) are not included.

As expected, the stability of the daily output has significantly improved with DCS. The standard deviation (*σ*) of the output for non‐DCS systems was less than 2%, and for DCS system was less than 1%. The percent of readings within 2*σ* is greater than 90% for all units. This result is supported by both ion chamber measurements and monitor chamber analysis. The results for all six systems are summarized in Table 2. Figures 2 and 3 are histograms of the output for non‐DCS and DCS systems, respectively, baselined at 100%. The output was tracked for over 60 months for each non‐DCS and over 30 months for the DCS systems. The effect of DCS is apparent in that the daily output ranged from ±3% in the older systems, and the introduction of DCS decreased the daily variation to less than 1%. The Radixact data were not included in Fig. 3 because of the limited data history available as compared to the other systems.

**Table 2:** Output stability. The standard deviation of output and (% within 2*σ* of mean) for all machines for which the data was available. An A1SL was used as an external ion chamber to measure output in solid water phantom. The monitor chamber signal is from the primary internal chamber (dose1)

	Non‐DCS 04	Non‐DCS 96	Non‐DCS 103	DCS 103	DCS 477	DCS 488	DCS RS
A1SL	N/A	N/A	0.43 (93.3%)	0.004 (94.1%)	0.56 (100%)	0.33 (92.9%)	0.34 (100%)
Monitor chamber	1.52 (97.3%)	1.30 (96.8%)	0.94 (96.8%)	0.004 (93.8%)	0.048 (97.4%)	0.061 (98.7%)	0.035 (100%)

**Figure 4:**
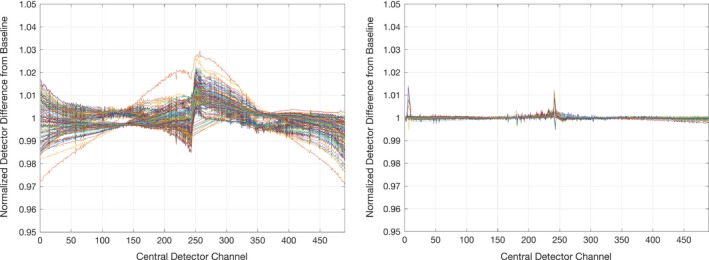
Comparisons of normalized exit detector ratios. Normalized exit detector ratios are illustrated as a surrogate for beam energy stability over time. The rotating target system (SN04, left) shows greater difference over all datasets compared to a fixed target (FTL) system (SN103, right) which shows less variation in profiles. Both datasets are displayed are on equal scales.

Energy stability also improved with the FTL and DCS. As shown in Table 3, the average RMS flatness value for rotating target systems ranged from 0.23% to 0.62%, while for fixed target systems the RMS flatness ranged from 0.04% to 0.09%. This difference is also illustrated in Fig. 4, where the normalized exit detector ratio is plotted over all datasets for a rotating target system (SN 04) and a fixed target system (SN 103). As a rotating target thins, the energy decreases and becomes more forward peaked, causing the shoulders of the exit detector ratio to drop^11^. The Radixact system appears to be as stable as the TomoHDA systems when considering RMS flatness. The average ion chamber PDD 20/10 ratio difference yielded similar variation in energy stability over time. However, the improved energy stability with the fixed target observed with the exit detector data is not appreciated with the ion chamber PDD 20/10 ratio due to lack of statistical power. The cone ratio data is the key driver in concluding the energy has improved with the FTL, as illustrated in Fig. 4.

**Table 3:** Energy stability. The mean and (number of data points) of exit detector RMS flatness and PDD 20/10 ratio for all machines

	Rotating 04	Rotating 96	Rotating 103	Fixed 103	Fixed 477	Fixed 488	Fixed RS
RMS flatness	0.62% (175)	0.23% (61)	0.59% (236)	0.09% (54)	0.07% (662)	0.09% (566)	0.04% (20)
PDD 20/10 ratio	N/A	N/A	0.18% (885)	N/A	0.49% (12)	0.72% (13)	0.04% (67)

The energy and output of both the clinical and Radixact systems were very stable during all extended plan durations tested. The CoV of the output and energy are summarized in Table 4. The CoV of the output at both depths (10 cm and 20 cm) was less than 0.2%, which is to be expected as both systems are using DCS. The CoV of the ratios of the 60 s averaged readings at the two depths demonstrate the energy stability during these extended deliveries.

**Table 4:** Extended duration delivery stability. The coefficient of variation (CoV) is reported for energy (ratio of 10 and 20 cm depth readings) and output (10 cm) during 20, 30 and 40 min. extended deliveries on Radixact and TomoHDA

	Plan duration [min]	CoV energy (%)	CoV 10 cm (%)
Radixact	20	0.062	0.079
	30	0.071	0.087
	40	0.099	0.109
TomoHDA	20	0.067	0.075
	30	0.059	0.077
	40	0.071	0.094

The coefficient of variation (CoV) is reported for energy (ratio of 10 and 20 cm depth readings) and output (10 cm) during 20, 30, and 40 min. Extended deliveries on Radixact and TomoHDA.

The service records from the machines are summarized in Table 5. The data do not cover the entire lifetime of the Hi‐Art units. The Hi‐Arts (SN 04, SN 96, and SN 103) had 10, 11, and 4 magnetron replacements in 42, 34, and 21 months, respectively. During that same time period, 5, 4, and 3 targets were replaced on the same Hi‐Art systems. With the introduction of the FTL, there have been no linac/target replacements on the TomoHDA systems. There have been no major system replacements on the Radixact system to date; however, it is important to note it that it does not have the same workload as the clinical systems. At the time of this manuscript, it is only typically running 8–16 hr per week.

**Table 5:** Major dosimetric component replacement history. The service record history for the Hi‐Art systems does not cover the complete duration of the output and energy study due to limited availability of older records

Unit [time period (mos.)]	Magnetron	Linac	Target
Hi‐Art SN 04 (42)	10	4	5
Hi‐Art SN 96 (34)	11	1	4
Hi‐Art 103(21)	4	0	3
Hi‐Art 103 (33), new target & linac design	6	0	1
TomoHDA 477 (31)	0	0	0
TomoHDA 488 (26)	1	0	0
Radixact (11)	0	0	0

The service record history does not cover the complete duration of this output and energy study due to limited availability of records for the Hi‐Art systems.

## 4 Discussion

Since its clinical inception in 2002, TomoTherapy has established itself as a versatile and robust treatment delivery system^12^, ^13^. TomoTherapy was conceived as a specialty machine, combining IMRT and IGRT^1^. Currently, TomoTherapy is used to treat a wide range of plans, from static gantry 3D radiation therapy to complex multitarget IMRT. This is due in part to the introduction of new features, but also because system reliability and stability have increased over time. As expected, the addition of the DCS has improved daily and intrafraction output stability significantly. The standard deviation in the monitor chamber daily output varies less than 0.5%. This stability allows for the delivery of longer complex treatment plans (e.g., TBI and total skin treatments^14^, ^15^) without the risk of treatment interruption. The dosimetric stability and system reliability have led to greater clinical utility.

Initial testing on the Radixact system has shown to be as stable as the current clinical TomoHDA systems. Sophisticated (and consequently long duration) plans can be delivered with the same level of dosimetric confidence. Moreover, these plans can be delivered faster due to the increased dose rate. With the nominal dose rate increased from 850 cGy/min to 1000 cGy/min, plans with the similar optimization parameters (jaw width, pitch, and modulation factor) can be delivered faster. This is because the gantry speed is set after the optimal fluence for the given parameter set is determined. The gantry speed is fixed in TomoTherapy. The minimum gantry period is set according to the longest MLC leaf open time needed to deliver the fluence at a given gantry angle. The range of gantry periods on Radixact is unchanged from the TomoHDA systems (11.8–60 s). With a higher dose rate, more fluence can be delivered at each angle allowing the gantry to speed up (to its maximum speed of 11.8 s per rotation). This is very important for high dose per fraction treatments. Moreover, the higher dose rate may allow for favorable changes in how TomoTherapy sequences the optimal fluence. This may result in certain combinations of pitch, modulation factor, and jaw size for a complex highly modulated high dose per fraction plan that would have reached upper gantry rotation limits with the lower dose rate. In addition to reduced beam‐on time, individual treatment times and patient throughput are improved if the machine is dosimetrically stable and reliable.

There is a sharp contrast in component replacement between the Hi‐Art and latest generation TomoTherapy treatment systems. Although we have highlighted the two most significant factors (DCS and FTL), numerous other incremental improvements were made across the gantry to improve overall system reliability. These improvements range from a newer magnetron design and RF feedback, waveguide design, and injector control assembly, all the way to temperature control systems and water flow rate management. All of these components can affect dosimetric stability, the lifetime of the major system components, and system uptime.

From a clinician's standpoint, it can sometimes be difficult to translate machine performance into patient care. Obviously any machine downtime is inconvenient for both patients and staff. Overtime, better hardware and software systems have (and continue) to address performance issues for all treatment delivery systems. Specifically, evaluating dosimetric stability is valuable because it has direct ties to patient plan delivery accuracy, as shown in longitudinal studies of patient plan QA^16^. Patient treatment interruption due to machine service has also been shown to affect outcome^17^. There are subtler effects as well. Extended downtime requires patient transfers and re‐planning which monopolizes dosimetry efforts, taking them away from treatment plans in progress. Major component replacements require significant post‐service quality assurance to confirm system consistency, which can delay other clinical duties by the physics team. Dosimetric instabilities can also increase the frequency of machine faults, which can lead to frustration by those delivering and receiving treatment. As such, the TomoHDA and Radixact systems represent an important advancement of the TomoTherapy system for both clinicians and patients.

## 5 Conclusion

TomoTherapy treatment systems have matured from a novel concept of IMRT delivery to fully integrated delivery systems in clinical practice. This long‐term review of the output and energy over multiple generations of the delivery systems has demonstrated high dosimetric stability. The fundamental redesign of the target and introduction of DCS has also led to improved reliability. Proof of equivalent energy and output stability on the Radixact system as monitored with a variety of metrics was presented, and found to be consistent with existing systems. Confidence in the overall dosimetric stability of the Radixact delivery system is important as potential advances of a higher dose rate system can only be realized with a stable system.

## Conflict of Interest

The work on the alpha Radixact research system is supported in part by a University of Wisconsin and Accuray™ research agreement.
